# Spatial genetic mapping links shared inflammatory bowel disease liability to adult immune–epithelial lesion contexts

**DOI:** 10.3389/fimmu.2026.1853177

**Published:** 2026-07-08

**Authors:** Guangyi Tao, Hanzhe Du, Jun Zhao, Zhonghe Zeng, Junwu Cui, Longming Lei

**Affiliations:** 1 Guangxi University of Chinese Medicine, Nanning, China; 2 The First Affiliated Hospital of Guangxi University of Chinese Medicine, Nanning, China

**Keywords:** adult spatial transcriptomics, Crohn’s disease, GWAS, immune–epithelial lesion context, inflammatory bowel disease, RT-qPCR, spatial genetic mapping, ulcerative colitis

## Abstract

**Background:**

Inflammatory bowel disease (IBD), including Crohn’s disease (CD) and ulcerative colitis (UC), shows marked clinical heterogeneity despite a shared immune-genetic background. The adult spatial contexts through which inherited IBD susceptibility is expressed remain unclear.

**Methods:**

We integrated GWAS summary statistics for overall IBD, CD, and UC with LDSC, stratified LDSC, LDSC-SEG, MAGMA, PoPS, and genetically informed spatial mapping (gsMap). Human genetic signals were projected onto the E16.5 mouse single-cell spatial atlas as an exploratory developmental reference, and adult disease-tissue spatial support was assessed across SCP2959 CD spatial sections and GSE189184 idiopathic UC inflamed Visium sections. PoPS-independent MAGMA-only module-score and FUSION-TWAS sensitivity analyses, together with targeted RT-qPCR in NCM460 epithelial cells and THP-1-derived macrophage-like cells were performed.

**Results:**

LDSC showed strong positive genetic correlations among overall IBD, CD, and UC, including overall IBD versus CD (r_g_ = 0.9458, P = 1.79 × 10^−8^), overall IBD versus UC (estimated r_g_ = 1.0907, P = 3.48 × 10^−6^), and CD versus UC (r_g_ = 0.9535, P = 0.0133). MAGMA and PoPS prioritized immune-inflammatory candidates, including IL23R, JAK2, STAT3, CCL2, NOD2, and HLA-region genes. Exploratory developmental gsMap showed nominal signals in gastrointestinal, liver, smooth-muscle, epidermal, and neural regions. In adult disease-tissue gsMap, overall IBD signals showed FDR-significant enrichment in SCP2959 CD immune regions (ACAT P = 3.52 × 10^−7^, q = 1.41 × 10^−6^), lamina propria (ACAT P = 2.75 × 10^−5^, q = 4.27 × 10^−5^), follicular clusters (ACAT P = 7.31 × 10^−7^, q = 1.10 × 10^−5^), and myeloid clusters (ACAT P = 6.67 × 10^−6^, q = 3.34 × 10^−5^). In GSE189184 idiopathic UC inflamed tissue, enrichment was observed in GWAS-independent immune-rich (ACAT P = 1.72 × 10^−5^, q = 1.38 × 10^−4^), structural/barrier (ACAT P = 7.52 × 10^−5^, q = 3.01 × 10^−4^), epithelial-mucosal (ACAT P = 9.08 × 10^−4^, q = 0.00182), inflammation-repair (ACAT P = 0.00140, q = 0.00224), and stromal-fibrotic domains (ACAT P = 0.00268, q = 0.00357). MAGMA-only module-score and FUSION-TWAS sensitivity analyses provided PoPS-independent support for the adult lesion-context interpretation. RT-qPCR showed that JAK2 knockdown reduced cytokine-induced CCL2 and CXCL8 by 52.6% and 36.9% and partially restored OCLN expression, while LPS induced IL1B, TNF, CCL2, and PYCARD in macrophage-like cells.

**Conclusion:**

Shared IBD genetic liability was most consistently linked to an adult immune–epithelial inflammatory lesion program involving immune-rich, epithelial-inflammatory, myeloid/follicular, lamina propria, structural/barrier, and remodeling-associated contexts. Developmental and subtype-weighted spatial signals, including neural-related signals in the embryonic reference, should be viewed as hypothesis-generating clues to developmental and neuroimmune programs rather than definitive subtype-specific mechanisms.

## 1 Introduction

Inflammatory bowel disease (IBD), mainly comprising Crohn’s disease (CD) and ulcerative colitis (UC), is a chronic immune-mediated disorder of the gastrointestinal tract with increasing global burden. CD and UC share recurrent intestinal inflammation, relapsing clinical courses, and substantial therapeutic challenges, but they differ in anatomical distribution, depth of inflammation, histopathological features, and complication patterns (1, 2). CD can affect any segment of the gastrointestinal tract and is often associated with transmural inflammation, stricturing, penetrating disease, and mesenteric remodeling, whereas UC is typically characterized by continuous mucosal inflammation of the colon and rectum (1, 3). These clinical differences suggest that IBD heterogeneity cannot be fully explained by systemic immune activation alone and requires a more precise understanding of how genetic susceptibility is expressed within disease-relevant tissue contexts.

Large-scale genetic studies have substantially advanced the understanding of IBD susceptibility. Genome-wide association studies have identified numerous loci shared across IBD subtypes, highlighting immune regulation, host–microbial interaction, cytokine signaling, epithelial barrier function, and innate host defense as central components of disease biology (4, 5). At the same time, CD and UC are not completely identical disease entities, and some genetic signals may show subtype-weighted effects (5, 6). Methods based on GWAS summary statistics, including LDSC, stratified LDSC, LDSC-SEG, MAGMA, and PoPS, provide complementary approaches for estimating shared genetic architecture, partitioning heritability across regulatory annotations, linking heritability to tissue-expression contexts, and prioritizing candidate genes (7–10). However, these approaches are limited in their ability to determine where inherited risk is expressed within spatially organized intestinal lesions.

Single-cell and spatial transcriptomic studies have shown that IBD lesions are organized through coordinated interactions among epithelial, immune, stromal, vascular, neural, and tissue-repair programs. In UC, single-cell analysis of human colonic tissue revealed extensive epithelial, immune, and stromal rewiring during inflammation (11). In CD, recent single-cell and spatial studies have highlighted fibrosis-associated networks and multicellular tissue niches in stricturing disease (12). These observations emphasize that disease-associated genetic signals should be interpreted not only at the level of genes or bulk tissues, but also within spatially resolved adult lesion environments. Spatial genetic mapping methods such as gsMap provide a framework for projecting GWAS signals onto spatial transcriptomic references, but developmental cross-species spatial references should be interpreted as exploratory discovery resources rather than substitutes for adult human disease-tissue spatial evidence (13, 14).

In this study, we integrated GWAS summary statistics for overall IBD, CD, and UC with genetic correlation analysis, regulatory annotation heritability enrichment, GTEx tissue-context enrichment, MAGMA gene-level association testing, PoPS candidate prioritization, gsMap-based developmental spatial discovery, adult disease-tissue spatial analyses and cross-section support, PoPS-independent MAGMA-only sensitivity analysis, and targeted RT-qPCR experiments. We aimed to distinguish shared IBD genetic liability from exploratory subtype-weighted spatial patterns and to determine whether IBD-associated genetic signals could be detected in adult CD and UC lesion contexts. By combining genetic, spatial, adult disease-tissue, and transcript-level experimental evidence, this study provides a cautious framework for linking IBD susceptibility to immune, epithelial-inflammatory, myeloid/follicular, lamina propria, and remodeling-associated adult intestinal lesion environments.

## 2 Methods

### 2.1 Study design and data sources

**Figure 1:**
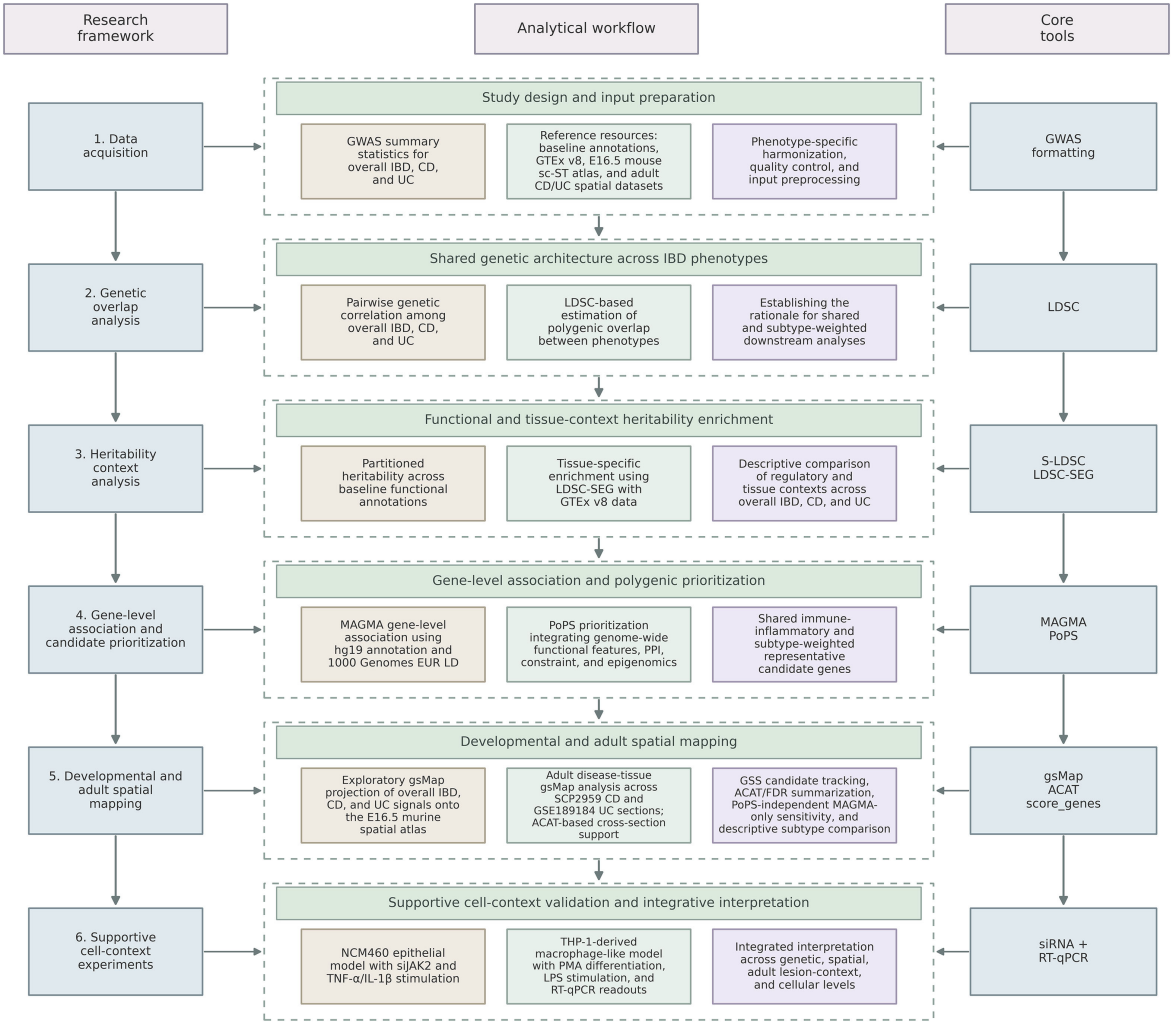
Flow chart of our study. GWAS, genome-wide association study; GTEx, genotype-tissue expression; LDSC, linkage disequilibrium score regression; S-LDSC, stratified LDSC; LDSC-SEG, LDSC specifically expressed genes; MAGMA, multi-marker analysis of GenoMic Annotation; PoPS, polygenic priority score; gsMap, genetically informed spatial mapping; GSS, gene-specific score; ACAT, aggregated Cauchy association test; RT-qPCR, reverse transcription quantitative polymerase chain reaction.

We obtained genome-wide association study (GWAS) summary statistics for overall inflammatory bowel disease (IBD), Crohn’s disease (CD), and ulcerative colitis (UC) from previously published large-scale European-ancestry cohorts and organized all analyses within an integrated framework designed to distinguish shared IBD genetic liability from exploratory subtype-weighted spatial patterns (**Figure 1**). The workflow included seven major components: acquisition and harmonization of phenotype-specific GWAS summary statistics and reference resources; pairwise genetic correlation analysis among overall IBD, CD, and UC; functional and tissue-context heritability enrichment using stratified LDSC and LDSC-SEG; gene-level association testing and knowledge-guided candidate prioritization using MAGMA and PoPS; exploratory developmental spatial mapping of GWAS signals onto the E16.5 murine single-cell spatial transcriptomic atlas using gsMap; adult disease-tissue gsMap analyses using adult CD and UC spatial transcriptomic references, together with PoPS-independent MAGMA-only sensitivity analysis; and supportive RT-qPCR experiments in epithelial and macrophage inflammatory cell models.

All bioinformatic analyses were conducted in R (version 4.4.1) and Python. Detailed information on GWAS datasets, transcriptomic and spatial references, software versions, analytical parameters, and data availability is provided in **Supplementary Tables 1**–**3**.

### 2.2 Reference data

For tissue-level heritability enrichment analyses, we used gene expression profiles from the Genotype-Tissue Expression (GTEx) project (v8), including 53 human tissues. For developmental spatial mapping, we used the E16.5 mouse single-cell spatial transcriptomic atlas implemented in the gsMap reference workflow. This atlas was used as an exploratory developmental reference rather than as an adult disease-tissue reference. For adult disease-tissue spatial support, we selected publicly available adult human intestinal spatial transcriptomic datasets with available spatial expression matrices, spot- or cell-level metadata, disease-relevant CD or UC tissue sections, and spatial annotations suitable for gsMap aggregation. Two datasets met these criteria and were included: SCP2959 adult CD spatial transcriptomic sections and GSE189184 adult UC Visium sections.

The SCP2959 adult CD reference included 20 public Visium sections grouped into five sample/specimen-prefix groups: V10A14-143, V10S15-054, V10S15-055, V11Y24-010, and V11Y24-011 (13). All sections that could be converted into gsMap-compatible spatial expression objects were included, and no section was excluded on the basis of gsMap significance. For GSE189184, because the original series contains both idiopathic ulcerative colitis and immune-checkpoint-therapy colitis samples, the primary adult UC analysis was restricted to seven sections whose GEO metadata indicated disease = ulcerative colitis, activity = inflamed, and immunotherapy = N/A: B4, B5, B8, B9, B12, B13, and C2 (14). These seven UC inflamed sections represented six GEO donor/specimen proxies because B8 and B9 shared the same GEO sample title, GI 6967. Healthy control, non-inflamed disease, and immune-checkpoint-therapy colitis sections were not included in the primary UC lesion-context analysis. Accordingly, these public spatial datasets were used to assess consistency across available tissue sections and donor/specimen proxies rather than to provide fully independent patient-level replication. Detailed metadata, section-level inclusion logic, donor/specimen-proxy information, evidence roles, and adult gsMap summaries are provided in **Supplementary Tables 1**-**S3**, **S6**.

### 2.3 Genetic correlation analysis

Pairwise genetic correlations among overall IBD, CD, and UC were estimated using LDSC. LDSC was used to evaluate the extent of shared polygenic architecture between phenotypes on the basis of GWAS summary statistics and linkage disequilibrium patterns across the genome. These analyses were used primarily to establish the degree of genetic overlap among IBD phenotypes and to justify downstream analyses of shared and subtype-weighted regulatory and spatial contexts.

### 2.4 Partitioned heritability analysis

To characterize the functional genomic architecture of IBD and its subtypes, we performed partitioned heritability analysis using S-LDSC. SNPs were annotated according to baseline functional genomic categories, including coding and regulatory regions, and annotation-specific LD scores were modeled to estimate heritability enrichment within each category. Enrichment profiles were generated for overall IBD, CD, and UC under the same analytical framework, and cross-phenotype comparisons were interpreted descriptively as relative enrichment patterns rather than as formal differential enrichment tests.

### 2.5 Tissue-specific heritability enrichment

To examine tissue context, we performed tissue-specific heritability enrichment analysis using LDSC-SEG in combination with GTEx v8 expression profiles across 53 tissues. LDSC-SEG evaluates whether trait heritability is enriched near genes specifically expressed in particular tissues. Trait-specific enrichment profiles were generated for overall IBD, CD, and UC, and relative differences in tissue enrichment patterns were interpreted descriptively because the three GWAS inputs were highly genetically correlated and may share components of the underlying study populations.

### 2.6 Gene-level association analysis and knowledge-guided candidate prioritization

Gene-level association analysis was performed using MAGMA. SNP-level association statistics were aggregated into gene-level signals using positional annotation based on the hg19 reference genome and LD information from the 1000 Genomes European reference panel. MAGMA results were used to identify genes showing strong gene-level association with overall IBD, CD, and UC. Genes reaching the Bonferroni-corrected MAGMA gene-level significance threshold were considered statistically significant, and complete MAGMA outputs are provided in **Supplementary Table 4**.

To contextualize candidate genes in a broader biological framework, we applied PoPS as a knowledge-guided candidate-prioritization layer. PoPS integrates multiple feature types, including GWAS association strength, protein–protein interaction information, evolutionary constraint, and epigenetic regulatory annotations, to assign genome-wide prioritization scores to genes. In the present study, PoPS was used to support candidate contextualization rather than as an independent validation method. Genes supported by both MAGMA evidence and high PoPS ranking were highlighted as representative candidates for downstream interpretation, whereas the robustness of adult spatial interpretation was further evaluated using a PoPS-independent MAGMA-only sensitivity analysis. Representative prioritized genes and complete PoPS results are provided in **Supplementary Table 4**.

### 2.7 Developmental spatial mapping using gsMap

To place IBD-associated genetic signals into an exploratory developmental spatial context, we applied gsMap (version 1.73.7) to integrate human GWAS summary statistics with the E16.5 murine single-cell spatial transcriptomic atlas spanning 25 annotated embryonic organs. The gsMap workflow includes graph-based extraction of spatial latent representations, cross-species ortholog mapping between mouse and human genes, smoothing of spatial gene expression profiles, calculation of gene-specific scores for individual spatial locations, construction of spatial LD scores using 50-kb gene windows, and spatial stratified LDSC to evaluate cell-level enrichment of genetic signals. Developmental gsMap analyses were performed separately for overall IBD, CD, and UC.

For each phenotype, organ-level association statistics were obtained by aggregating cell-level P values within each annotated embryonic organ using the Cauchy combination test. Raw organ-level Cauchy P values were reported together with multiple-testing-adjusted summaries across the 25 organs within each phenotype and across organ–phenotype combinations. Organ-level signals with unadjusted P < 0.05 were described as nominal or exploratory unless they survived multiple-testing correction. Because this analysis involved cross-species projection onto an embryonic reference rather than adult disease tissue, developmental gsMap results were interpreted as hypothesis-generating and were not treated as confirmatory evidence of adult IBD mechanisms.

### 2.8 Gene-specific score extraction and developmental candidate tracking

For each phenotype, genes were ranked according to gsMap-derived GSS values within the developmental spatial reference. Top-ranked GSS genes were retained as descriptive candidate-tracking outputs and summarized in **Supplementary Table 5** together with organ-level Cauchy combination results. These GSS-ranked genes were used to trace spatially prioritized developmental candidates in relation to MAGMA, PoPS, adult spatial evidence, and experimental feasibility, but they were not treated as an independent validation layer.

Because the developmental gsMap analysis was exploratory, downstream interpretation did not rely on GSS-ranked genes alone. Instead, adult disease-tissue gsMap cross-section evidence and PoPS-independent MAGMA-only sensitivity analysis were used to evaluate whether the main spatial interpretation could be supported in adult CD and UC lesion contexts.

### 2.9 Adult disease-tissue gsMap analysis and cross-section support

To evaluate whether IBD-associated genetic signals could be detected in adult intestinal disease contexts, we applied gsMap to adult spatial transcriptomic references from SCP2959 adult CD tissue and GSE189184 adult UC tissue. For SCP2959, region-level and cluster-level annotations were used for within-section aggregation. For GSE189184, primary analyses used GWAS-independent biological program domains defined independently of MAGMA, PoPS, or candidate-gene prioritization. Candidate-gene-informed domains were retained only as supportive sensitivity analyses, and unsupervised k-means domains were used as a label-independent sensitivity analysis without assuming identical biological meaning across sections.

For each adult spatial section, gsMap was used to calculate spot-level or cell-level spatial association statistics for overall IBD, CD, and UC GWAS inputs. Annotation-level evidence within each section was summarized by aggregating spot-level or cell-level P values within predefined spatial domains using the Cauchy combination test (15), and cross-section evidence for each annotation was summarized using ACAT. Adult gsMap results were reported using the number of contributing sections, minimum Cauchy P value, median Cauchy P value, ACAT P value, and Benjamini–Hochberg FDR-adjusted q values, with FDR correction performed within each dataset, GWAS input, and annotation scheme. Because the public spatial references contained multiple sections per donor/specimen proxy, section-level ACAT summaries were interpreted as cross-section consistency rather than independent patient-level replication. The main adult spatial analysis focused on overall IBD GWAS enrichment in adult CD and idiopathic UC inflamed lesion contexts, whereas CD- and UC-specific GWAS inputs were analyzed as comparative and descriptive evidence.

### 2.10 PoPS-independent MAGMA-only and FUSION-TWAS sensitivity analyses

To assess whether the adult spatial interpretation depended on PoPS knowledge-guided prioritization, we performed a PoPS-independent MAGMA-only sensitivity analysis. Candidate genes were selected from MAGMA gene-level association results without incorporating PoPS scores. Two MAGMA-only gene sets were generated for each phenotype: genes reaching the Bonferroni-corrected MAGMA gene-level significance threshold and the top 50 genes ranked by MAGMA gene-level P value. Gene identifiers were converted to gene symbols and matched to the adult spatial expression objects from SCP2959 and GSE189184, and the number of retained genes in each adult reference was recorded.

For each adult spatial dataset, MAGMA-only module scores were calculated from normalized spatial expression matrices after restricting each gene set to matched genes. In SCP2959, module scores were summarized across region- and cluster-level annotations; in GSE189184, module scores were summarized across curated program-domain and k-means spatial-domain annotations. Within each section, module scores in a given spatial annotation were compared with those in the remaining spots or cells, and the direction of median score differences was recorded. Cross-section evidence was summarized using ACAT, with FDR-adjusted summaries reported in **Supplementary Table 8**. Because large spatial datasets can yield very small spot-level P values, MAGMA-only sensitivity results were interpreted primarily according to cross-section consistency, direction of median score differences, and concordance with adult gsMap enrichment patterns rather than exact P-value magnitude alone.

As an additional PoPS-independent transcriptomic prioritization sensitivity analysis, we performed FUSION-TWAS using selected GTEx v8 expression-weight panels relevant to intestinal and immune contexts, including colon sigmoid, colon transverse, small intestine terminal ileum, and whole blood. FUSION was used because it prioritizes genes by integrating GWAS summary statistics with genetically predicted gene expression, providing a transcriptome-based prioritization layer distinct from PoPS knowledge-guided feature scoring. For each GWAS input, FDR correction was performed within each trait–tissue panel and across all four selected tissues within the same trait. FUSION-TWAS results were interpreted as supportive transcriptomic prioritization evidence rather than as independent proof of genetic causality or adult spatial localization. Complete FUSION-TWAS outputs and summary counts are provided in **Supplementary Table 9**.

### 2.11 Cell culture, inflammatory stimulation, siRNA transfection, and RT-qPCR

Human normal colonic epithelial NCM460 cells (catalogue no. CL-0299) and human monocytic THP-1 cells (catalogue no. CL-0233) were obtained from Procell Life Science & Technology Co., Ltd. (Wuhan, China). NCM460 cells were cultured in Dulbecco’s modified Eagle medium (DMEM; Procell, catalogue no. PM150210), and THP-1 cells were cultured in RPMI-1640 medium (Procell, catalogue no. PM150110). Both media were supplemented with 10% fetal bovine serum (FBS; ExCell Bio, Shanghai, China; catalogue no. FND500) and 1% penicillin–streptomycin (Biosharp, Hefei, China; catalogue no. BL505A). Cells were maintained at 37 °C in a humidified incubator with 5% CO_2_. JAK2 was selected for targeted epithelial knockdown because it was supported by gene-level prioritization and linked the shared IBD genetic signal to epithelial inflammatory responses. For the epithelial inflammatory model, NCM460 cells were treated with recombinant human TNF-α (10 ng/mL; Sino Biological, Beijing, China; catalogue no. 10602-HNAE) and recombinant human IL-1β (10 ng/mL; Sino Biological; catalogue no. 10139-HNAE) for 24 h. For the macrophage inflammatory model, THP-1 cells were differentiated with phorbol 12-myristate 13-acetate (PMA, 100 ng/mL; Beyotime, Shanghai, China; catalogue no. S1809) for 24 h, rested for 24 h in fresh medium, and then stimulated with lipopolysaccharide (LPS, 1 μg/mL; Beyotime; catalogue no. S1732) for 24 h.

For JAK2 knockdown, NCM460 cells were seeded in six-well plates and transfected at approximately 50%–70% confluence with a small interfering RNA targeting human JAK2 (siJAK2) or a non-targeting negative-control siRNA (siNC), both synthesized by GenePharma Co., Ltd. (Shanghai, China). Transfection was performed using Lipo8000 Transfection Reagent (Beyotime; catalogue no. C0533) according to the manufacturer’s protocol. At 48 h after transfection, cells were treated with TNF-α/IL-1β as described above. The NCM460 groups were siNC, siJAK2, siNC + TNF-α/IL-1β, and siJAK2 + TNF-α/IL-1β. Total RNA was extracted using the FastPure Cell/Tissue Total RNA Isolation Kit (Vazyme, Nanjing, China; catalogue no. RC101), and RNA concentration and purity were assessed using a NanoDrop 2000 spectrophotometer. cDNA was synthesized from 1 μg of total RNA using HiScript III RT SuperMix for qPCR (+gDNA wiper) (Vazyme; catalogue no. R323). RT-qPCR was performed using ChamQ Universal SYBR qPCR Master Mix (Vazyme; catalogue no. Q711) on a QuantStudio 5 Real-Time PCR System. In NCM460 cells, JAK2, CCL2, CXCL8, and OCLN were measured to assess knockdown efficiency, inflammatory response, and epithelial barrier-related transcriptional changes. In THP-1-derived macrophage-like cells, CCL2, IL1B, TNF, and PYCARD were measured to assess myeloid inflammatory activation. GAPDH was used as the reference gene. Three independent biological replicates were included for each group, and each biological replicate was measured in technical duplicate. Technical Ct replicates were averaged before relative quantification using the 2^−ΔΔCt^ method. Primer sequences, siRNA sequences, raw Ct values, and plotting data are provided in **Supplementary Table 7**.

### 2.12 Statistical analysis

Unless otherwise specified, statistical analyses were performed within the native frameworks of the corresponding methods. For LDSC, S-LDSC, LDSC-SEG, MAGMA, PoPS, and gsMap analyses, method-specific statistical outputs were used as described above. For gsMap analyses, cell-level or spot-level P values were aggregated within annotated spatial domains using the Cauchy combination test, and cross-section evidence in adult spatial datasets was summarized using ACAT. For PoPS-independent sensitivity analyses, MAGMA-only gene sets were defined without using PoPS scores, and adult module-score results were interpreted as sensitivity support rather than as independent genetic association tests. RT-qPCR data from three independent biological replicates were presented as mean ± standard error of the mean (SEM). Statistical comparisons were performed on ΔCt values normalized to GAPDH using two-sided Welch’s t-tests. For NCM460 cells, predefined comparisons included siNC versus siJAK2, siNC versus siNC + TNF-α/IL-1β, and siNC + TNF-α/IL-1β versus siJAK2 + TNF-α/IL-1β. For THP-1-derived macrophage-like cells, control and LPS-stimulated groups were compared. A two-sided P < 0.05 was considered statistically significant. Exact P values are provided in **Supplementary Table 7**.

## 3 Results

### 3.1 Shared genetic architecture across IBD phenotypes

Pairwise LDSC analysis showed strong positive genetic correlations among overall inflammatory bowel disease (IBD), Crohn’s disease (CD), and ulcerative colitis (UC) (**Table 1**). The strongest correlation was observed between overall IBD and UC (r_g_ = 1.0907, P = 3.48 × 10^−6^), followed by overall IBD and CD (r_g_ = 0.9458, P = 1.79 × 10^−8^). CD and UC also showed a positive genetic correlation (r_g_ = 0.9535, P = 0.0133). These results indicate that IBD and its two major subtypes share a broadly overlapping polygenic background, providing a rationale for subsequent analyses aimed at resolving regulatory, tissue, and spatial contexts of subtype-weighted genetic signals. The estimate slightly exceeding 1 for overall IBD versus UC likely reflects sampling variability in unconstrained LDSC estimation and should not be interpreted as exceeding the theoretical upper bound of genetic correlation.

**Table 1:** Genetic correlations among overall IBD and its major subtypes estimated by LDSC.

Trait pairs	LDSC P	LDSC r_g_ (SE)
Overall IBD & Ulcerative Colitis (UC)	3.48×10^−6^	1.0907 (0.2351)
Overall IBD & Crohn’s Disease (CD)	1.79×10^−8^	0.9458 (0.1679)
Ulcerative Colitis (UC) & Crohn’s Disease (CD)	0.0133	0.9535 (0.3853)

LDSC, Linkage Disequilibrium Score Regression; rg, genetic correlation; SE, standard error.

### 3.2 Functional regulatory and tissue-context heritability enrichment

**Figure 2:**
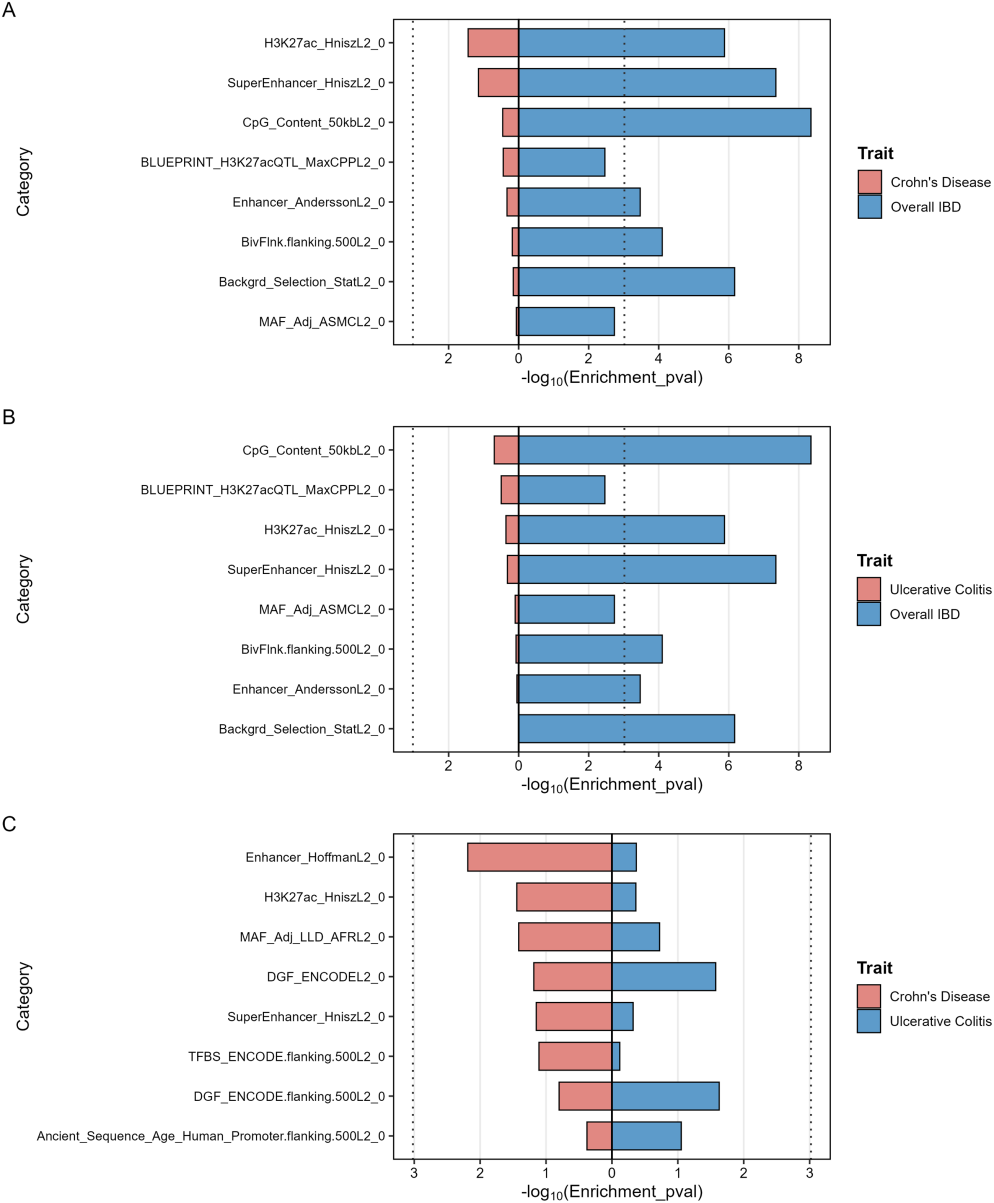
Partitioned heritability enrichment across functional genomic annotations. Analyses were performed using stratified LDSC. Panels **(A, B)** compare overall IBD with CD and UC, respectively, and highlight enrichment in conserved regulatory annotations, including CpG content regions, super-enhancers, and H3K27ac-marked elements. Panel **(C)** shows relative differences in annotation enrichment profiles between CD and UC, including enhancer- and DNase hypersensitive site-related annotations. Dashed lines indicate the adjusted significance thresholds.

We next used stratified LDSC and LDSC-SEG to characterize regulatory annotation and GTEx tissue-context enrichment across the three genetically correlated IBD phenotypes. In stratified LDSC analysis, overall IBD showed enrichment in conserved regulatory annotations, including CpG content regions, super-enhancers, and H3K27ac-marked regions (**Figures 2A, B**). Descriptive comparison of enrichment profiles suggested that CD had relatively stronger signals in enhancer-associated annotations, including Enhancer_HoffmanL2_0 and H3K27ac-marked regions, whereas UC showed relatively stronger enrichment in DNase I hypersensitivity-related annotations, represented by DGF_ENCODEL2_0 (**Figure 2C**). Because these comparisons were based on highly correlated GWAS inputs, they were interpreted as descriptive subtype-weighted patterns rather than formal differential enrichment tests.

**Figure 3:**
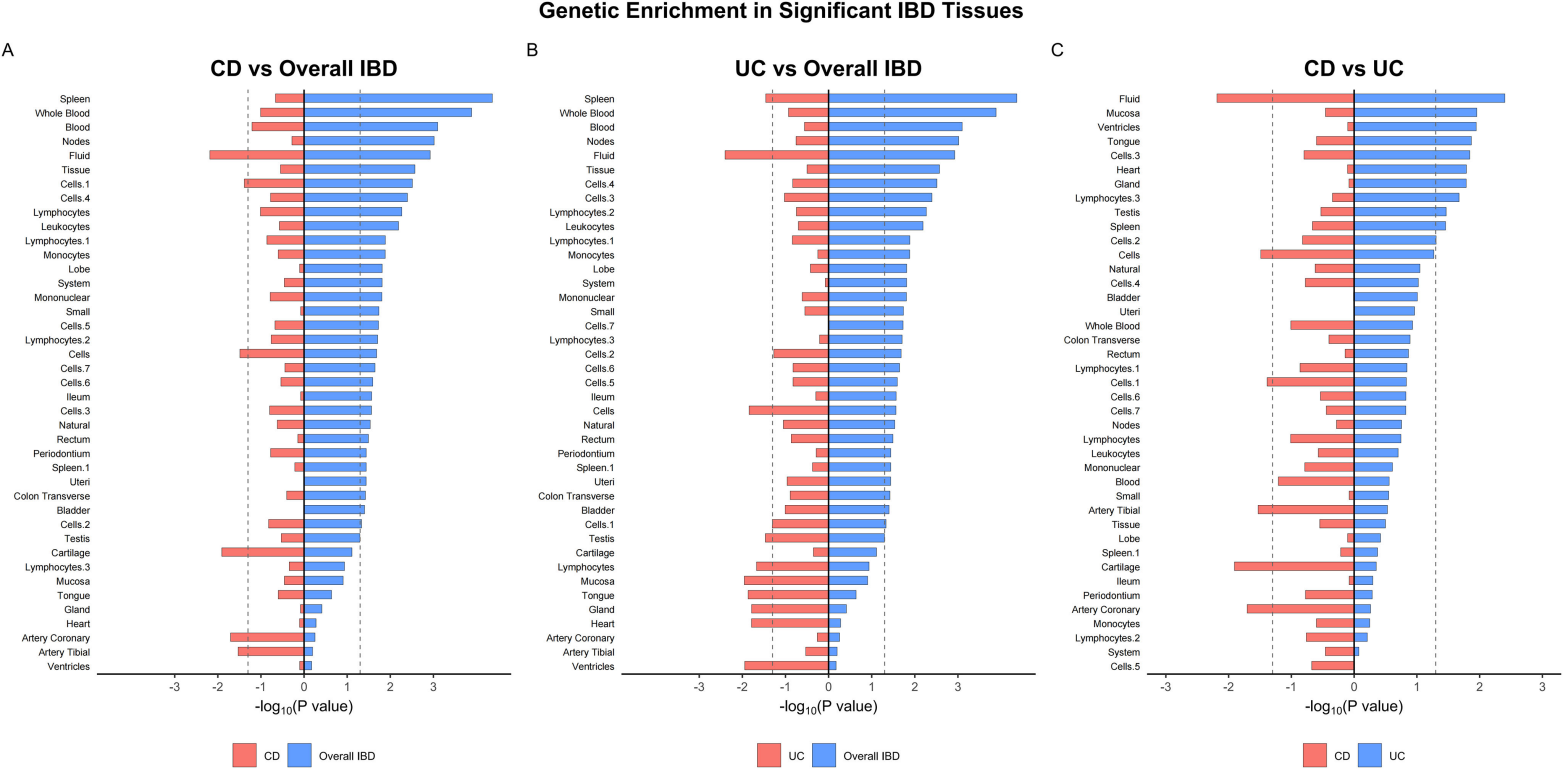
Tissue-specific heritability enrichment across GTEx tissues. The x-axis represents −log10(P value). Panels **(A, B)** show enrichment patterns for overall IBD relative to CD and UC, respectively, highlighting shared signals in immune-related tissues such as spleen, whole blood, and lymphocyte-associated tissues. Panel **(C)** compares trait-specific enrichment profiles between CD and UC and suggests relative subtype-weighted differences in tissue context. These results were interpreted as tissue-context enrichment rather than direct mechanistic validation.

LDSC-SEG analysis further showed that overall IBD, CD, and UC shared prominent enrichment in immune-related GTEx tissues, including spleen, whole blood, and lymphocyte-associated tissues (**Figures 3A, B**). Descriptive comparison of trait-specific LDSC-SEG profiles suggested additional tissue-context differences, with CD showing relatively stronger enrichment in fluid- and mucosa-related tissues and UC showing stronger enrichment in colon transverse and rectum (**Figure 3C**). These findings support a shared immune genetic background with possible differences in tissue-context weighting, but they do not establish fixed subtype-specific mechanisms. Therefore, these results were used to motivate downstream spatial analyses in developmental and adult disease-tissue references.

### 3.3 Gene-level association and knowledge-guided candidate prioritization

**Figure 4:**
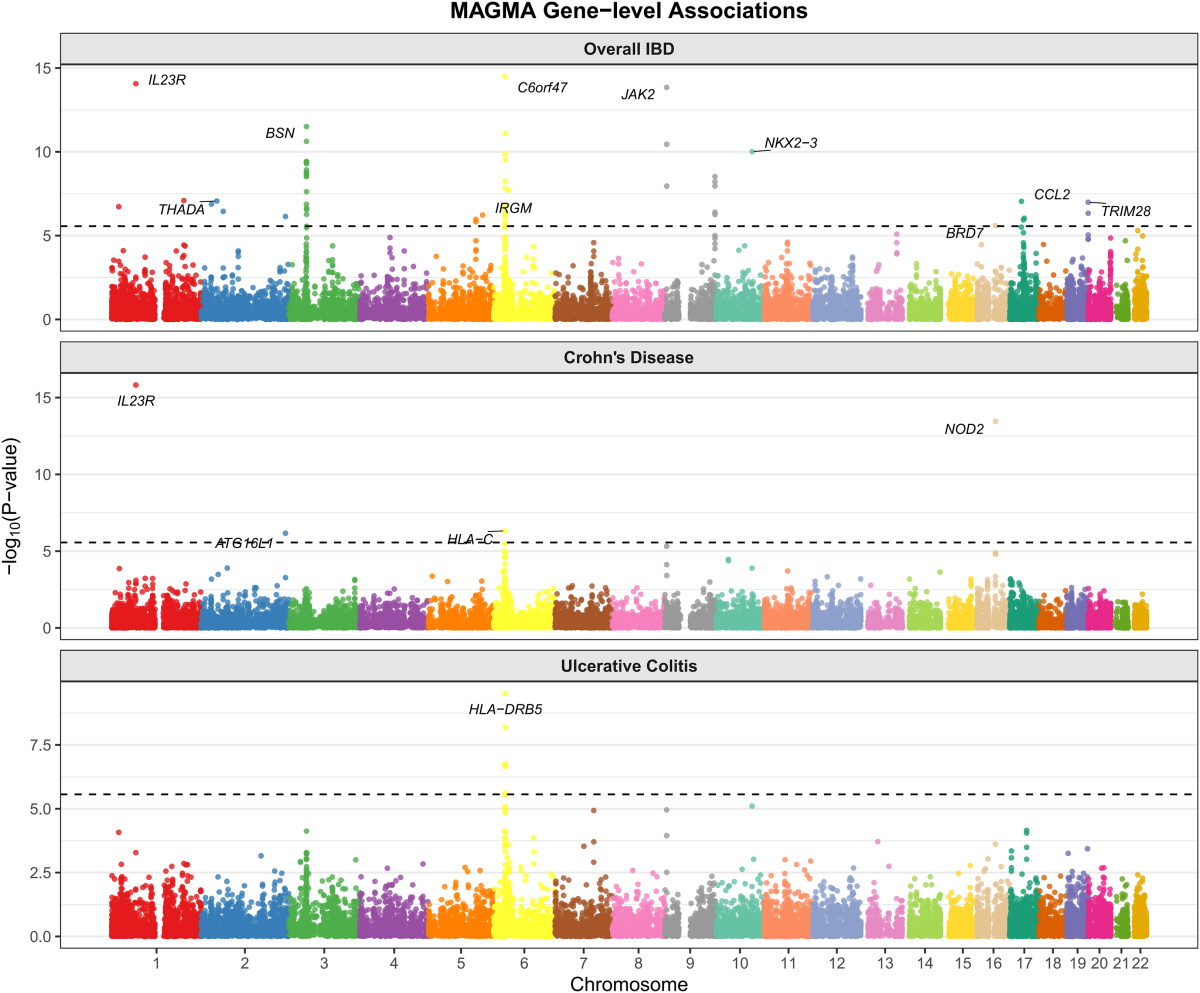
MAGMA gene-level association analysis for overall IBD and its subtypes. The figure shows gene-level Manhattan plots for overall IBD, CD, and UC. The y-axis represents −log10(P value) for gene-level associations. The top panel highlights representative significant loci in overall IBD, including IL23R and JAK2. The middle panel shows CD-associated signals, including NOD2 and ATG16L1. The bottom panel identifies UC-associated signals in the MHC region, including HLA-DRB5. Horizontal dashed lines indicate the Bonferroni-corrected gene-level significance threshold (P < 2.5 × 10^−6^).

Gene-level association analysis using MAGMA identified multiple risk genes associated with overall IBD and its subtypes (**Figure 4**). In overall IBD, several loci reached the Bonferroni-corrected gene-level significance threshold, including IL23R, JAK2, STAT3, and CCL2. Subtype-stratified analyses highlighted additional subtype-weighted signals, with NOD2 showing a prominent association in CD and HLA-DRB5 emerging as a representative UC-associated signal in the MHC region.

To complement gene-level association testing, we applied the Polygenic Priority Score (PoPS) framework as a knowledge-guided prioritization layer. Genes with strong MAGMA evidence were frequently also prioritized by PoPS (**Table 2**). IL23R received the highest PoPS score in overall IBD (PoPS = 5.377) and also ranked highly in CD (PoPS = 3.368). Other highly prioritized genes included JAK2 (PoPS = 3.663 in overall IBD; 2.945 in UC), STAT3 (PoPS = 2.738 in overall IBD), and CCL2 (PoPS = 2.445 in overall IBD). In CD, NOD2 showed strong prioritization (PoPS = 2.959), whereas NKX2–3 remained among the higher-ranked candidates in UC. Because PoPS incorporates external biological annotations and prior knowledge, these rankings were interpreted as complementary evidence for candidate prioritization rather than as independent mechanistic validation. Subsequent spatial analyses therefore focused on the overall genetic signals of each phenotype, while gene-level and PoPS results were used to contextualize convergent candidate genes for downstream interpretation and experimental follow-up.

**Table 2:** Representative gene-level association signals and PoPS-supported candidate genes in IBD and its subtypes.

Gene symbol	MAGMA significance	PoPS score (Overall IBD)	PoPS score (CD)	PoPS score (UC)
IL23R	Overall IBD, CD	5.377	3.368	1.880
JAK2	Overall IBD	3.663	1.292	2.945
STAT3	Overall IBD	2.738	0.945	0.798
CCL2	Overall IBD	2.445	1.252	0.243
NKX2-3	Overall IBD	2.355	1.142	2.160
FOSL2	Overall IBD	1.558	0.939	0.052
NOD2	CD	1.335	2.959	0.806
IRGM	Overall IBD	1.293	-0.130	-0.024
CCND3	Overall IBD	0.953	0.197	0.908
PDLIM4	Overall IBD	0.759	0.303	0.223
CARD9	Overall IBD	0.712	0.244	0.217
RHOA	Overall IBD	0.634	0.310	0.079
INSL6	Overall IBD	0.494	0.079	0.396
P4HA2	Overall IBD	0.434	-0.027	0.105
BRD7	Overall IBD	0.397	-0.019	-0.124
GPX1	Overall IBD	0.356	0.204	0.243
TRIM28	Overall IBD	0.334	0.084	-0.202
UBA7	Overall IBD	0.304	-0.055	-0.354
INAVA	Overall IBD	0.292	0.072	0.116
OTUD3	Overall IBD	0.286	0.114	0.140

This table summarizes representative genes supported by MAGMA gene-level association analysis and PoPS-based candidate prioritization. “MAGMA Significance” indicates the phenotype(s) in which the gene reached the Bonferroni-corrected gene-level significance threshold (P < 2.5 × 10−6). PoPS scores were used as a complementary prioritization layer and were not interpreted as independent validation of biological mechanisms. Only representative top-ranked genes are shown; the complete list of scored genes is provided in **Supplementary Table 4**.

### 3.4 Developmental gsMap discovery in the E16.5 mouse embryo spatial atlas

**Figure 5:**
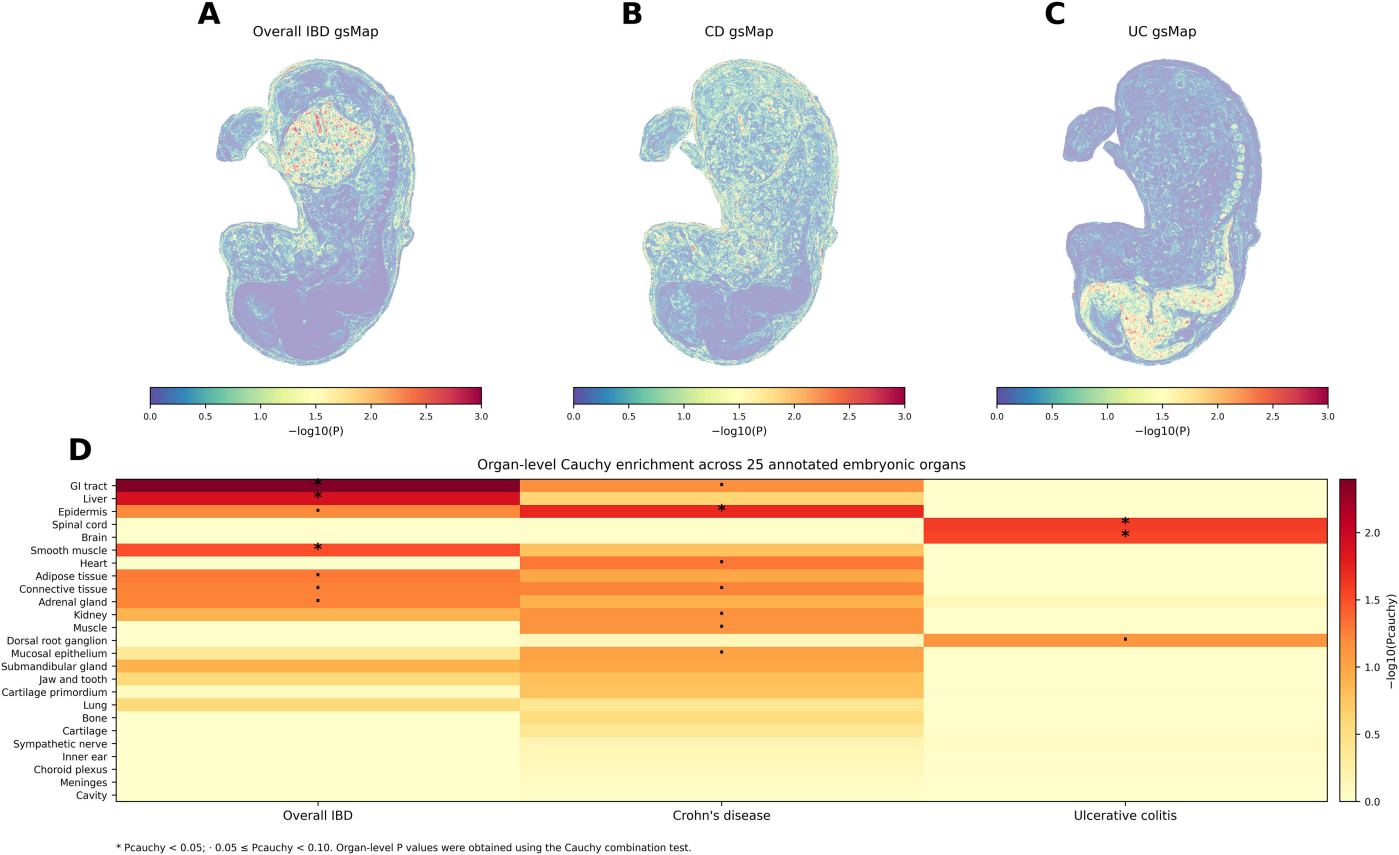
Developmental gsMap discovery in the E16.5 mouse embryonic spatial transcriptomic atlas. **(A–C)** Cell-level gsMap spatial enrichment maps for overall inflammatory bowel disease (IBD), Crohn’s disease (CD), and ulcerative colitis (UC) projected onto the E16.5 mouse single-cell spatial transcriptomic atlas. Colors indicate −log10(P) values from cell-level gsMap association statistics. **(D)** Organ-level enrichment summary across 25 annotated embryonic organs using the Cauchy combination test. Asterisks indicate nominal enrichment (P*_Cauchy_* < 0.05), and dots indicate trend-level evidence (0.05 ≤ P*_Cauchy_* < 0.10). These developmental gsMap results were interpreted as exploratory and hypothesis-generating rather than as direct evidence of adult disease mechanisms. All developmental organ-level findings, including neural-related UC signals, represent exploratory cross-species projections and do not establish adult disease-tissue localization or subtype-specific mechanisms.

Following gene-level association testing and candidate prioritization, we applied gsMap to the E16.5 mouse single-cell spatial transcriptomic atlas as an exploratory developmental spatial reference. Cell-level gsMap statistics were aggregated across 25 annotated embryonic organs using the Cauchy combination test, and organ-level enrichment profiles were compared across overall IBD, CD, and UC (**Figure 5**; **Supplementary Table 5**). For overall IBD, the strongest nominal organ-level signals were observed in the gastrointestinal tract (P_Cauchy_ = 0.0040), liver (P_Cauchy_ = 0.0128), and smooth muscle (P_Cauchy_ = 0.0318). CD showed a nominal developmental signal in epidermis (P_Cauchy_ = 0.0193), with weaker trend-level signals in connective tissue, gastrointestinal tract, kidney, muscle, and mucosal epithelium. UC showed nominal enrichment in neural tissues, including spinal cord (P_Cauchy_ = 0.0254) and brain (P_Cauchy_ = 0.0284), with a trend-level signal in dorsal root ganglion (P_Cauchy_ = 0.0764). These embryonic neural-related findings were nominal and exploratory and were not interpreted as evidence of an adult UC-specific neural mechanism.

These developmental gsMap results were interpreted as hypothesis-generating rather than as validation of adult IBD mechanisms. The overall IBD signal projected preferentially onto gastrointestinal, hepatic/metabolic, smooth-muscle, and mesenchymal or structural developmental contexts, whereas CD and UC showed weaker and partially divergent developmental patterns. Because this analysis involved cross-species projection onto an embryonic reference and multiple organ-level comparisons, nominal organ-level signals were not considered evidence of subtype-specific adult pathology. Gene-specific score-ranked genes were therefore retained as descriptive candidate-tracking outputs in **Supplementary Table 5**, whereas the main biological interpretation was based on adult disease-tissue gsMap cross-section support.

### 3.5 Adult disease-tissue gsMap cross-section support and PoPS-independent sensitivity analyses

**Figure 6:**
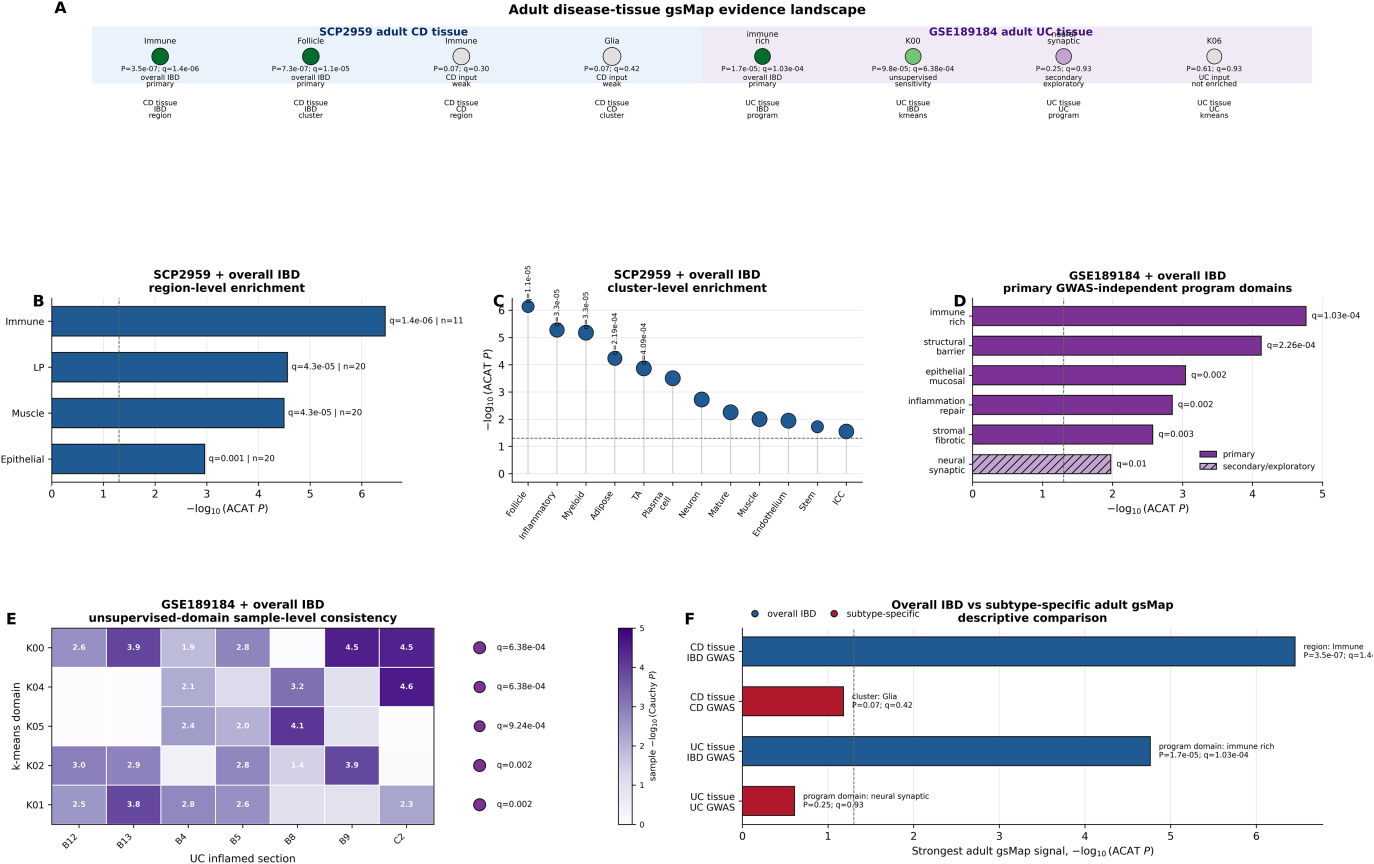
Adult disease-tissue gsMap cross-section support for shared IBD genetic liability in CD and UC lesions. **(A)** Overview of adult disease-tissue gsMap analyses across SCP2959 adult CD spatial transcriptomic sections and GSE189184 adult UC Visium sections. Cell- or spot-level gsMap statistics were aggregated within predefined spatial annotations using the Cauchy combination test, and cross-section evidence was summarized using ACAT with Benjamini–Hochberg FDR correction. **(B)** Region-level enrichment of overall IBD GWAS signals in SCP2959 adult CD tissue. **(C)** Cluster-level enrichment of overall IBD GWAS signals in SCP2959 adult CD tissue. **(D)** Program-domain enrichment of overall IBD GWAS signals in GSE189184 adult UC tissue. The neural/synaptic domain was retained as a secondary exploratory domain and was not used to define the primary adult lesion-context conclusion or to infer a UC-specific neural mechanism. **(E)** Unsupervised k-means spatial-domain sensitivity analysis in GSE189184. **(F)** Comparison of overall IBD and subtype-specific GWAS inputs in adult disease-tissue gsMap analyses. Overall IBD GWAS signals showed robust adult spatial enrichment in both CD and UC lesions, whereas CD- and UC-specific GWAS inputs produced weaker enrichment in the available adult spatial references.

We next applied gsMap to adult disease-tissue spatial datasets to assess cross-section support for the localization of IBD-associated genetic signals in inflamed adult intestinal contexts. The analysis included 20 SCP2959 adult CD sections grouped into five specimen-prefix groups and seven primary GSE189184 idiopathic UC inflamed sections representing six donor/specimen proxies. Therefore, ACAT-derived summaries quantify consistency across the available sections rather than fully independent patient-level replication. Cell- or spot-level gsMap statistics were aggregated within predefined spatial annotations using the Cauchy combination test, and cross-section evidence was summarized using ACAT with Benjamini–Hochberg FDR correction (**Figure 6**; **Supplementary Table 6**). In SCP2959 adult CD tissue, overall IBD GWAS signals showed consistent enrichment across immune-rich and tissue-remodeling compartments. At the region level, enrichment was strongest in immune regions (n = 11 sections; minimum P_Cauchy_ = 3.69 × 10^−8^; median P_Cauchy_ = 9.04 × 10^−6^; ACAT P = 3.52 × 10^−7^; q = 1.41 × 10^−6^), followed by lamina propria (ACAT P = 2.75 × 10^−5^; q = 4.27 × 10^−5^), muscle (ACAT P = 3.21 × 10^−5^; q = 4.27 × 10^−5^), and epithelial regions (ACAT P = 0.00110; q = 0.00110). Cluster-level analysis further localized the overall IBD signal to follicular regions (ACAT P = 7.31 × 10^−7^; q = 1.10 × 10^−5^), inflammatory clusters (ACAT P = 5.32 × 10^−6^; q = 3.34 × 10^−5^), and myeloid clusters (ACAT P = 6.67 × 10^−6^; q = 3.34 × 10^−5^), with additional FDR-supported signals in epithelial, plasma cell, vascular, stromal, and muscle-associated annotations.

In GSE189184 adult UC tissue, the primary analysis was restricted to seven idiopathic UC inflamed sections and GWAS-independent biological program domains. Overall IBD GWAS signals showed FDR-significant enrichment in immune-rich domains (n = 7 sections; minimum P_Cauchy_ = 2.97 × 10^−6^; median P_Cauchy_ = 2.11 × 10^−4^; ACAT P = 1.72 × 10^−5^; q = 1.38 × 10^−4^), followed by structural/barrier-related domains (ACAT P = 7.52 × 10^−5^; q = 3.01 × 10^−4^), epithelial-mucosal domains (ACAT P = 9.08 × 10^−4^; q = 0.00182), adult inflammation-repair domains (ACAT P = 0.00140; q = 0.00224), and stromal-fibrotic domains (ACAT P = 0.00268; q = 0.00357). The UC neural/synaptic domain also showed FDR-supported enrichment for overall IBD GWAS signals (ACAT P = 0.0106; q = 0.0121), but it was treated as a secondary exploratory domain and was not considered the dominant adult UC signal. Low-signal domains were retained in the supplementary output as reference annotations but were not biologically interpreted. Candidate-gene-informed domains, including the shared immune MAGMA/PoPS program, showed concordant enrichment for overall IBD but were treated as supportive sensitivity analyses because they incorporated MAGMA/PoPS-derived information.

To assess whether the adult spatial interpretation depended on PoPS knowledge-guided prioritization, we performed a PoPS-independent MAGMA-only module-score sensitivity analysis. Genes were selected using MAGMA Bonferroni significance or the top 50 genes ranked by MAGMA gene-level P value, without using PoPS scores. Most MAGMA-only genes were retained in the adult spatial expression datasets, including 48 of 50 overall IBD MAGMA-top50 genes in SCP2959 and 47 of 50 in GSE189184, as well as 119 of 124 overall IBD MAGMA-Bonferroni genes in both adult references. These matched genes included IL23R, JAK2, CARD9, CCL2, HLA-DRA, HLA-DRB1, and NKX2-3. MAGMA-only module-score analyses provided PoPS-independent expression-localization support in immune, follicular, myeloid, lamina propria, epithelial, and structural/barrier-related adult spatial contexts, broadly consistent with the adult gsMap results (**Supplementary Table 8**). Together, these results support the conclusion that the main adult disease-tissue signal reflected shared IBD immune and epithelial-inflammatory lesion contexts and was not solely attributable to PoPS-based prioritization. We further performed a PoPS-independent FUSION-TWAS sensitivity analysis using four selected GTEx v8 tissue panels relevant to intestinal and immune biology. Across colon sigmoid, colon transverse, small intestine terminal ileum, and whole blood, FUSION tested 4,132–8,615 transcriptomic models per trait–tissue panel. Overall IBD showed the strongest transcriptomic prioritization signal, with 142 FDR-significant gene–tissue associations after FDR correction across all four selected tissues within the trait. In contrast, CD and UC each showed five FDR-significant gene–tissue associations under the same correction scheme. These results provided an additional PoPS-independent transcriptomic prioritization layer and supported the interpretation that the most robust signal was associated with shared overall IBD genetic liability rather than strong subtype-specific separation (**Supplementary Table 9**).

### 3.6 Supportive RT-qPCR evidence for epithelial and macrophage inflammatory responses

To provide supportive transcript-level validation for the adult disease-tissue spatial findings, we performed RT-qPCR in two complementary inflammatory cell models: an epithelial model using NCM460 cells with siRNA-mediated JAK2 knockdown and TNF-α/IL-1β stimulation, and a macrophage inflammatory model using LPS-stimulated THP-1-derived macrophage-like cells. JAK2 was selected because it was supported by gene-level prioritization and provided a tractable link between shared IBD genetic signals and epithelial inflammatory responses. CCL2, CXCL8, and OCLN were used as epithelial inflammatory and barrier-associated readouts, whereas IL1B, TNF, CCL2, and PYCARD were used to support the macrophage/myeloid inflammatory context highlighted by the adult spatial analyses.

**Figure 7:**
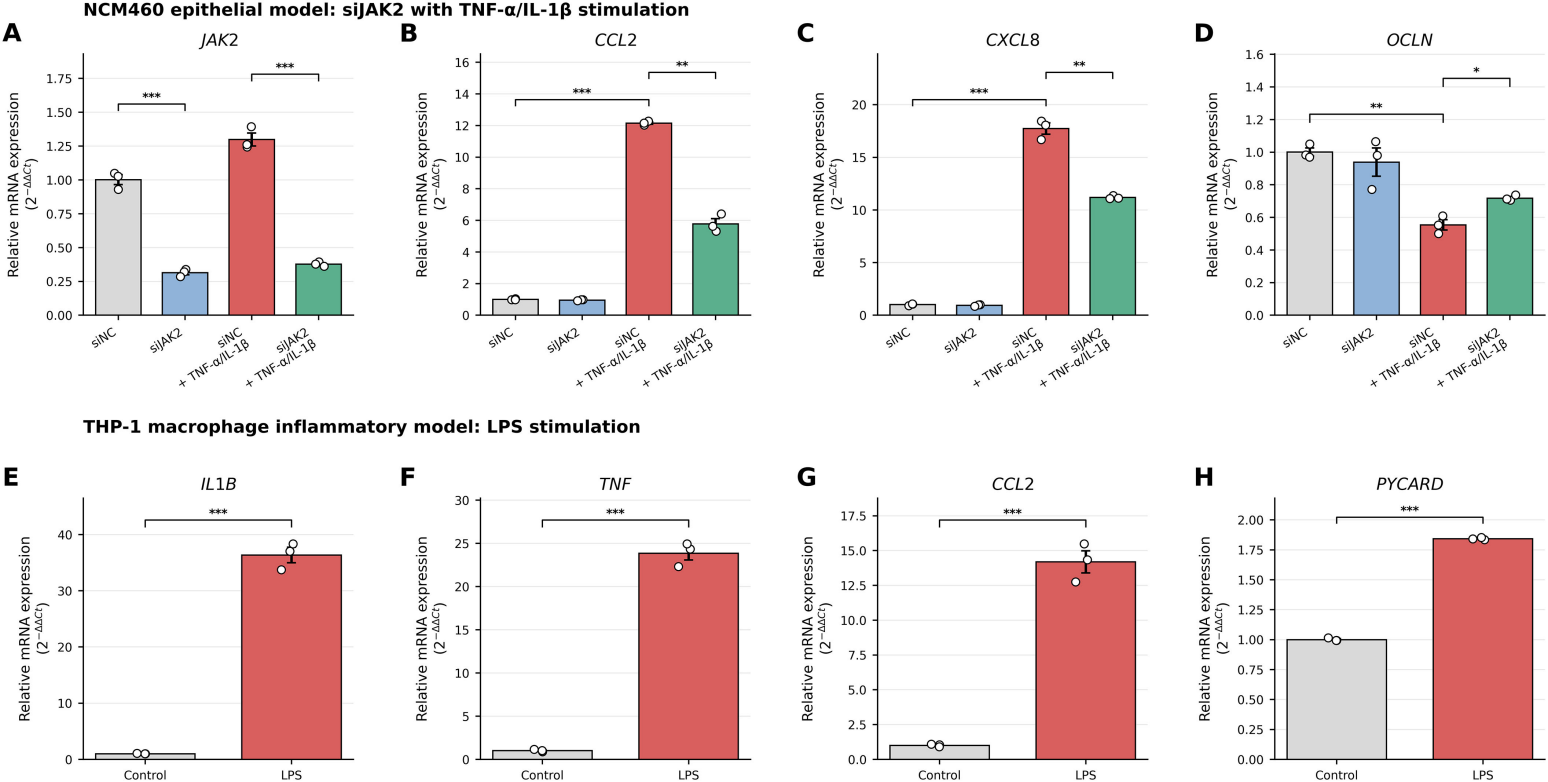
Supportive RT-qPCR validation in epithelial and macrophage inflammatory models. **(A–D)** Relative mRNA expression of JAK2, CCL2, CXCL8, and OCLN in NCM460 epithelial cells after siRNA-mediated JAK2 knockdown with or without TNF-α/IL-1β stimulation. **(E–H)** Relative mRNA expression of IL1B, TNF, CCL2, and PYCARD in THP-1 macrophage-like cells after LPS stimulation. Bars indicate mean ± SEM from three independent biological replicates, and dots represent individual biological replicates. Technical duplicates were averaged before analysis. Relative expression was calculated using the 2^-ΔΔCt method after normalization to GAPDH. Statistical significance was assessed on ΔCt values using two-sided Welch’s t-tests. Only predefined pairwise comparisons are shown. *P < 0.05, **P < 0.01, ***P < 0.001. The raw Ct values and plotting data are provided in **Supplementary Table 7**. Exact P values are provided in **Supplementary Table 7**.

In NCM460 cells, siRNA-mediated knockdown efficiently reduced JAK2 mRNA expression. Compared with the siNC group, JAK2 expression was reduced to approximately 31% after siJAK2 transfection (**Figure 7A**). TNF-α/IL-1β stimulation induced a marked epithelial inflammatory response, with CCL2 and CXCL8 expression increased by approximately 12.2-fold and 17.7-fold, respectively, compared with the unstimulated siNC group (**Figures 7B, C**). In contrast, the epithelial barrier-associated gene OCLN was decreased to approximately 55% of the control level after inflammatory stimulation (**Figure 7D**). In stimulated NCM460 cells, siJAK2 reduced CCL2 expression by approximately 52.6% and CXCL8 expression by approximately 36.9% compared with the siNC + TNF-α/IL-1β group, while OCLN expression was partially restored (**Figures 7B–D**).

In THP-1-derived macrophage-like cells, LPS stimulation induced a robust inflammatory transcriptional response. IL1B, TNF, and CCL2 were increased by approximately 36.3-fold, 23.8-fold, and 14.1-fold, respectively, compared with untreated control cells (**Figures 7E–G**). PYCARD showed a moderate but consistent increase of approximately 1.84-fold after LPS stimulation (**Figure 7H**). Together, these RT-qPCR experiments provide supportive cell-level transcript evidence for epithelial inflammatory/barrier responses and macrophage inflammatory programs highlighted by the adult disease-tissue spatial analyses, while remaining supportive rather than definitive mechanistic validation.

## 4 Discussion

In this study, we integrated GWAS summary statistics, heritability partitioning, gene-level association analysis, spatial genetic mapping, adult disease-tissue spatial analyses, and targeted RT-qPCR experiments to examine how inherited susceptibility to IBD may be expressed across regulatory, tissue, spatial, and cellular contexts. The most consistent finding was that overall IBD, CD, and UC shared a substantial genetic background, and that this shared genetic liability was preferentially detected in adult intestinal lesion contexts enriched for immune, epithelial-inflammatory, myeloid/follicular, lamina propria, and tissue-remodeling features. These findings support a spatial genetic model in which inherited IBD risk is not expressed as a single isolated pathway, but as an adult immune–epithelial inflammatory lesion program. In this model, genetic liability is linked to coordinated immune and myeloid activation, epithelial chemokine induction, barrier-related epithelial changes, follicular/lamina propria organization, and stromal-remodeling or repair-associated tissue contexts. This interpretation is consistent with large-scale IBD genetic studies showing extensive overlap between IBD subtypes (4, 5), and with single-cell and spatial transcriptomic studies indicating that intestinal inflammation is organized through coordinated immune, epithelial, stromal, and repair programs rather than isolated cell populations alone (12, 13). Although several analyses suggested relative differences between CD and UC, these patterns were insufficient to define fixed subtype-specific mechanisms. Instead, our findings indicate that shared IBD genetic susceptibility was most consistently detected across the available adult spatial sections within inflammatory and epithelial-remodeling lesion environments, whereas subtype-weighted developmental signals provide additional hypotheses for future investigation.

The high genetic correlations among overall IBD, CD, and UC provide an important context for interpreting the downstream analyses. CD and UC differ in anatomical distribution, inflammatory depth, histopathological features, complications, and clinical behavior, but many susceptibility loci and immune-regulatory pathways are shared across the IBD spectrum (4, 6). In this setting, S-LDSC and LDSC-SEG provided complementary information about the regulatory annotation and GTEx tissue-expression contexts of inherited risk. LDSC-based methods are widely used to estimate genetic correlation and partition SNP heritability across functional annotations or specifically expressed gene sets (7, 9). In our analysis, immune-related tissue enrichment across IBD phenotypes reinforced the central role of immune regulation in IBD susceptibility, whereas relative differences in regulatory annotations and GTEx tissue-context profiles suggested that overlapping genetic risk may be differentially weighted across mucosal, systemic, and colonic contexts. However, because the GWAS inputs for overall IBD, CD, and UC were highly correlated, cross-phenotype enrichment differences should be interpreted descriptively rather than as formal differential genetic mechanisms. Differences in GWAS power, phenotype definition, cohort composition, and reference annotations may also contribute to apparent subtype-weighted patterns.

The MAGMA and PoPS analyses provided a gene-level layer for interpreting these polygenic findings. MAGMA aggregates variant-level association statistics into gene-level signals, whereas PoPS prioritizes genes by leveraging polygenic enrichment across diverse gene features (10, 11). Several representative genes prioritized in our analysis, including IL23R, JAK2, STAT3, CCL2, CARD9, NOD2, HLA-DRA, HLA-DRB1, HLA-DRB5, and NKX2-3, are related to immune activation, cytokine signaling, antigen presentation, epithelial inflammation, innate host defense, and host–microbial interaction. These candidates are consistent with established pathway-level interpretations of IBD genetics, in which cytokine signaling, microbial sensing, epithelial barrier biology, and immune regulation converge across susceptibility loci (6, 16). However, PoPS incorporates external biological annotations, protein interaction information, evolutionary constraint, and regulatory features. Therefore, PoPS results are useful for candidate prioritization and biological contextualization, but they should not be interpreted as independent validation of disease mechanisms. To reduce this dependency, we performed a MAGMA-only sensitivity analysis that did not use PoPS scores. The recovery of immune, epithelial, lamina propria, follicular, myeloid, and structural/barrier-related adult spatial contexts in this analysis suggests that the main adult lesion-context interpretation was not solely driven by PoPS knowledge-based prioritization. Consistently, an additional FUSION-TWAS sensitivity analysis using intestinal and immune GTEx v8 expression-weight panels provided a transcriptome-based prioritization layer independent of PoPS and again showed stronger support for overall IBD than for subtype-specific CD or UC signals.

The developmental gsMap analysis was designed to provide an exploratory spatial projection of IBD-associated genetic signals rather than direct validation in adult disease tissue. gsMap links GWAS summary statistics with spatial transcriptomic references to identify spatial locations or cell states associated with complex trait heritability (17). The E16.5 mouse spatial atlas provides a valuable developmental map of organogenesis and tissue programs, but it does not represent adult human intestinal inflammation (18). Therefore, organ-level signals in gastrointestinal, hepatic/metabolic, smooth-muscle, epidermal, mesenchymal, or neural developmental regions should be interpreted as hypothesis-generating projections of genetic liability onto developmental transcriptional programs, not as direct evidence of adult CD or UC pathology. This distinction is particularly important for the UC neural/synaptic signal. Enteric neuroimmune communication is biologically relevant to intestinal homeostasis and inflammation (19, 20). However, the nominal neural enrichment observed in the developmental reference was not the dominant adult UC spatial signal in our analysis. Adult UC enrichment was more consistently captured by GWAS-independent immune-rich, epithelial-mucosal, inflammation-repair, structural/barrier, and stromal-fibrotic program domains. Thus, neural-related developmental signals may generate hypotheses about neuroimmune or developmental programs in intestinal inflammation, but further validation in adult human enteric nervous system, epithelial–neural, or neuroimmune spatial datasets would be required before assigning them a subtype-specific role in UC.

The adult disease-tissue spatial analyses were central for defining this spatial genetic model. In SCP2959 adult CD tissue, overall IBD genetic signals were enriched in immune regions, lamina propria, epithelial regions, and muscle-related compartments, and were further localized to follicular, inflammatory, and myeloid clusters. In GSE189184 adult UC tissue, overall IBD signals were captured by GWAS-independent immune-rich, epithelial-mucosal, inflammation-repair, structural/barrier-related, and stromal-fibrotic program domains, whereas candidate-gene-informed domains were treated as supportive sensitivity analyses. These results suggest that shared IBD genetic liability is spatially concentrated in lesion environments where immune activation, epithelial injury, myeloid inflammation, barrier remodeling, and tissue repair occur together. Therefore, the adult spatial evidence does not simply add another validation dataset; it provides the disease-tissue layer of the framework by showing where inherited risk is most consistently expressed within human intestinal lesions. This interpretation is consistent with recent adult single-cell and spatial transcriptomic studies showing that intestinal lesions are organized by multicellular immune, epithelial, stromal, and remodeling niches rather than by isolated immune-cell activation alone (13, 14). Importantly, this framework also clarifies the relationship between shared and subtype-weighted signals. The adult spatial analyses did not support a simple fixed separation between CD and UC. Instead, overall IBD GWAS signals produced the most consistent adult lesion-context enrichment, whereas subtype-specific CD and UC GWAS inputs showed weaker enrichment in the available adult references. Thus, subtype-weighted signals may still reflect biologically meaningful differences in disease presentation, tissue distribution, or developmental programs, but the strongest adult spatial evidence supports a shared immune–epithelial inflammatory lesion program. In this sense, the proposed spatial genetic framework does not argue that CD and UC are identical; rather, it suggests that their inherited susceptibility converges on a shared adult lesion architecture, while subtype-weighted signals remain secondary and require additional validation.

The RT-qPCR experiments provided targeted transcript-level support for two experimentally tractable components of the spatially inferred adult lesion program. The first component was a JAK2-linked epithelial chemokine/barrier-response axis. In NCM460 epithelial cells, TNF-α/IL-1β stimulation induced CCL2 and CXCL8 expression and reduced OCLN expression, whereas JAK2 knockdown attenuated chemokine induction and partially restored OCLN expression. This result links a genetically prioritized inflammatory signaling gene, JAK2, to epithelial chemokine production and barrier-related transcriptional change, which corresponds to the epithelial-mucosal and structural/barrier domains detected in the adult spatial analyses. This pattern is biologically consistent with studies showing that inflammatory cytokines can reshape intestinal epithelial function, chemokine production, and tight-junction barrier integrity (21, 22). The use of JAK2 knockdown is also compatible with the broader therapeutic relevance of JAK signaling in UC, although our experiment tested a targeted epithelial transcript response rather than drug efficacy (23). The second experimentally supported component was a macrophage inflammatory context. In THP-1-derived macrophage-like cells, LPS stimulation increased IL1B, TNF, CCL2, and PYCARD expression, supporting a myeloid inflammatory state aligned with the immune-rich and myeloid-related adult spatial signals. THP-1-derived macrophage-like cells are widely used as an *in vitro* model for immune-modulation studies, and inflammasome-related pathways, including PYCARD/ASC-associated signaling, have recognized relevance to mucosal inflammation in IBD (24, 25). Taken together, these experiments do not prove genetic causality or reconstruct the full epithelial–immune–stromal lesion microenvironment. However, they provide focused transcript-level evidence that two cellular components predicted by the spatial genetic framework—epithelial inflammatory/barrier response and macrophage inflammatory activation—are biologically responsive in relevant inflammatory cell models.

Several limitations should be considered. First, the genetic analyses were based on GWAS summary statistics from predominantly European-ancestry cohorts, which may limit generalizability to more diverse populations and reinforces the need for broader ancestry representation in future IBD genetic studies (26). Second, the overall IBD, CD, and UC GWAS inputs were highly correlated, so cross-phenotype comparisons should be interpreted as descriptive subtype-weighted patterns rather than formal differential genetic mechanisms. Third, the developmental gsMap analysis relied on cross-species projection onto an E16.5 mouse embryo atlas. It was hypothesis-generating and cannot provide adult human disease-tissue evidence. Fourth, the adult spatial analyses included 20 SCP2959 sections grouped into five specimen-prefix groups and seven primary GSE189184 idiopathic UC inflamed sections representing six donor/specimen proxies. Therefore, ACAT summaries should be interpreted as cross-section consistency in the available public references rather than as independent patient-level replication. In addition, the two adult spatial datasets differed in disease subtype, sample composition, annotation strategy, and analytical resolution, which limits direct CD–UC symmetry. Fifth, the GSE189184 program-domain annotations were generated in this study and require further validation, although k-means domain analysis and MAGMA-only sensitivity analyses were used to reduce dependence on curated labels and PoPS-based prioritization. Finally, the RT-qPCR experiments were performed in commercial cell-line models with three independent biological replicates and measured transcript-level responses only. Because mRNA abundance does not always directly predict protein abundance or pathway activity, additional protein-level assays, organoid models, co-culture systems, and patient-derived functional validation will be needed to establish causal mechanisms (27, 28).

## 5 Conclusion

In conclusion, this study provides a spatial genetic framework for interpreting how inherited IBD susceptibility is expressed within tissue contexts. By integrating GWAS-derived genetic evidence with regulatory annotation analysis, tissue-context heritability enrichment, gene-level prioritization, developmental spatial mapping, adult disease-tissue spatial analyses and cross-section support, and targeted RT-qPCR assays, we found that shared IBD genetic liability is most consistently linked to an adult immune–epithelial inflammatory lesion program. This program involves immune-rich, epithelial-inflammatory, myeloid/follicular, lamina propria, structural/barrier, and remodeling-associated contexts, rather than a single isolated pathway or a fixed subtype-specific mechanism. Developmental and subtype-weighted spatial signals, including neural-related signals in the embryonic reference, should be viewed as hypothesis-generating clues to developmental and neuroimmune programs rather than definitive subtype-specific mechanisms. Overall, this framework connects IBD genetic susceptibility with adult intestinal lesion architecture and identifies candidate spatial programs for future mechanistic validation.

## Data Availability

The original contributions presented in the study are included in the article/**Supplementary Material**. Further inquiries can be directed to the corresponding authors.
